# Cushing disease due to a somatic *USP8* mutation in a patient with evolving pituitary hormone deficiencies due to a germline *GH1* splicing variant

**DOI:** 10.20945/2359-3997000000428

**Published:** 2022-01-14

**Authors:** Julia Haddad Labello, Anna Flávia Figueredo Benedetti, Bruna Viscardi Azevedo, Alexander Augusto de Lima Jorge, Valter Angelo Sperling Cescato, Sergio Rosemberg, Fernando Pereira Frasseto, Ivo Jorge Prado Arnhold, Luciani Renata Silveira de Carvalho

**Affiliations:** 1 Faculdade de Medicina da Universidade de São Paulo Disciplina de Endocrinologia e Metabologia Hospital das Clínicas São Paulo SP Brasil Unidade de Endocrinologia do Desenvolvimento, Laboratório de Hormônios e Genética Molecular/LIM42, Hospital das Clínicas, Disciplina de Endocrinologia e Metabologia, Faculdade de Medicina da Universidade de São Paulo, São Paulo, SP, Brasil; 2 Universidade de São Paulo Faculdade de Medicina Laboratório de Sequenciamento em Larga Escala São Paulo SP Brasil Laboratório de Sequenciamento em Larga Escala (SELA), Faculdade de Medicina, Universidade de São Paulo, São Paulo, SP, Brasil; 3 Universidade de São Paulo Faculdade de Medicina Disciplina de Endocrinologia e Metabologia São Paulo SP Brasil Unidade de Endocrinologia Genética/LIM25, Hospital das Clínicas, Disciplina de Endocrinologia e Metabologia, Faculdade de Medicina, Universidade de São Paulo, São Paulo, SP, Brasil; 4 Universidade de São Paulo Faculdade de Medicina Instituto de Psiquiatria São Paulo SP Brasil Neurocirurgia Funcional, Instituto de Psiquiatria, Faculdade de Medicina, Universidade de São Paulo, São Paulo, SP, Brasil; 5 Universidade de São Paulo Faculdade de Medicina Hospital das Clínicas São Paulo SP Brasil Departamento de Patologia, Hospital das Clínicas, Faculdade de Medicina, Universidade de São Paulo, São Paulo, SP, Brasil

## Abstract

We present the unique case of an adult Brazilian woman with severe short stature due to growth hormone deficiency with a heterozygous G to T substitution in the donor splice site of intron 3 of the growth hormone 1 ( *GH1* ) gene (c.291+1G>T). In this autosomal dominant form of growth hormone deficiency (type II), exon 3 skipping results in expression of the 17.5 kDa isoform of growth hormone, which has a dominant negative effect over the bioactive isoform, is retained in the endoplasmic reticulum, disrupts the Golgi apparatus, and impairs the secretion of other pituitary hormones in addition to growth hormone deficiency. This mechanism led to the progression of central hypothyroidism in the same patient. After 5 years of growth and thyroid hormone replacement, at the age of 33, laboratory evaluation for increased weight gain revealed high serum and urine cortisol concentrations, which could not be suppressed with dexamethasone. Magnetic resonance imaging of the sella turcica detected a pituitary macroadenoma, which was surgically removed. Histological examination confirmed an adrenocorticotropic hormone (ACTH)-secreting pituitary macroadenoma. A ubiquitin-specific peptidase 8 ( *USP8* ) somatic pathogenic variant (c.2159C>G/p.Pro720Arg) was found in the tumor. In conclusion, we report progression of isolated growth hormone deficiency due to a germline *GH1* variant to combined pituitary hormone deficiency followed by hypercortisolism due to an ACTH-secreting macroadenoma with a somatic variant in *USP8* in the same patient. Genetic studies allowed etiologic diagnosis and prognosis of this unique case.

## INTRODUCTION

Hypopituitarism can be defined by the deficiency of one or more pituitary hormones. Growth hormone deficiency (GHD) can be isolated (IGHD) or associated with combined pituitary hormone deficiencies (CPHD). The first gene associated with GHD was the growth hormone 1 ( *GH1* ) ( 1 ). IGHD has been classified according to the inheritance pattern into three subcategories. Type I (IA and IB) is recessive: Type IA is associated with severe short stature with the absence of circulating GH and can sometimes involve a poor response to recombinant human growth hormone (rhGH) treatment due to autoantibody production. Type IB is characterized by low but detectable levels of GH, short stature, delayed bone age, and a good response to rhGH treatment without antibody formation; this type is associated with missense, nonsense, frameshift, and splice site mutations in the *GH1* and growth hormone release hormone receptor ( *GHRHR* ) genes. Type II is autosomal dominant and mainly caused by mutations within the first 6 base pairs (bp) of intervening sequences 3 (5’-IVS-3), which result in a mis-splicing at the messenger RNA (mRNA) level and the subsequent loss of E3, producing a 17.5 kDa GH isoform. From the clinical point of view, severe short stature (< -4.5 standard deviation score [SDS]) in type II is not present in all affected individuals, indicating that in some forms of IGHD type II, growth failure is less severe than one might expect ( 2 ). Type III is X-linked recessive and GHD can be associated with agammaglobulinemia. This classification of GHD can now be subdivided according to the gene defect due to the recent identification of genetic heterogeneity within each GHD subtype.

Over the past four decades, defects in the *GH1* and *GHRHR* genes have been associated with IGHD. In addition to these classic genes, IGHD has also been associated with a mutation in the *POU1F1* ( 3 ), *RNPC3* ( 4 ), *IARS2* ( 5 ), *GLI2* ( 6 ), *HESX1* , and *SOX3* ( 7 ) genes. However, in the vast majority of patients, the molecular cause remains undefined.

The type of GHD and frequency of *GH1* variants identified in different studies depends on ethnicity and population selection criteria, such as inbreeding frequency, severity of short stature, and GH secretory status ( 8 , 9 ). In some populations, such as the Dutch and German, type II is the most frequent genetic form of GHD ( 10 ). However, previous studies of molecular genetics in a Brazilian cohort of patients with GHD followed at the Developmental Endocrinology Unit of the *Hospital das Clínicas* of the University of São Paulo Medical School (HCFMUSP) identified only patients with *GH1* deletions and biallelic *GHRHR* mutations ( 11 , 12 ). In 2014, Lido and cols. ( 13 ) identified pathogenic *GH1* defects in 9 patients with IGHD, eight with the autosomal recessive form, and one with the autosomal dominant form. The latter patient had a heterozygous nucleotide substitution in the first nucleotide of intron 3 (c.291+1G>T) ( 13 ).

In the present study, we describe more detailed clinical characteristics of this patient, progression from IGHD to CPHD, and the development of adrenocorticotropic hormone (ACTH)-dependent Cushing disease due to a somatic pituitary ubiquitin-specific peptidase 8 ( *USP8* ) variant.

## CASE REPORT

**Figure 1:**
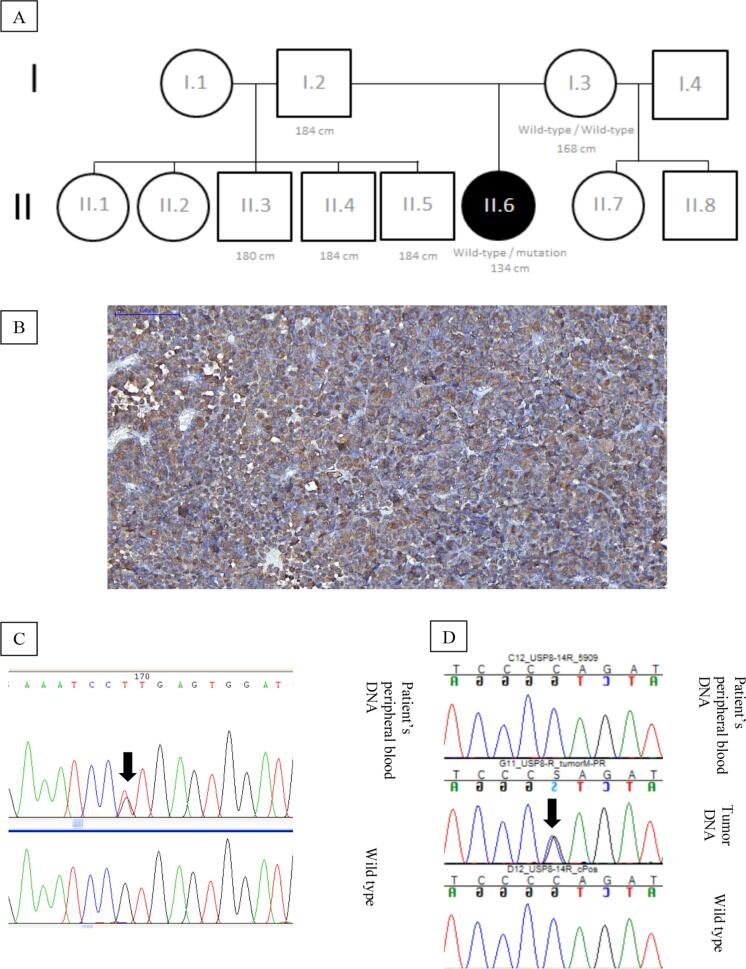
( **A** ) Genogram of the family of the patient with the growth hormone 1 ( *GH1* ) c.291 + 1G> T mutation in heterozygosis; informed height measurements: I.2, II.3, II.4, II. ( **B** ) Immunohistochemistry analyses (×200 magnification) of the resected pituitary tumor revealed positive and diffuse adrenocorticotropic hormone (ACTH) staining. ( **C** ) *GH1* Sanger sequencing showing c.291+1G>T germline *GH1* variant in intron 3 (black arrow) from the peripheral blood DNA of our index patient (above) and wild type chromatogram (below). ( **D** ) *USP8* sanger sequencing: first, no mutation is shown in our patient’s peripheral blood DNA, proving that it is not a germline mutation; second, the c.2159C>G/p.Pro720Arg mutation in the tumor DNA (black arrow); third, wild type chromatogram.

The index patient is a Brazilian woman with short stature, who was diagnosed with GHD when she was 8 years old but was not treated with rhGH replacement therapy due to financial problems. She had spontaneous menarche at the age of 15 years, with irregular cycles since menarche, and she began using oral contraceptives at the age of 21 to regularize her menstrual cycles. In 2007, when she was 28 years old, she sought medical attention because of short stature and obesity (height = 134 cm, *z* -score final height = -4.5, body mass index [BMI] = 30.4 kg/m^2^, Tanner stage M4P4). No consanguinity or similar cases were found in her family. Her mother’s height was 168 cm ( *z* -score +1.0), her father’s height was 184 cm ( *z* -score +1.4) (informed measurements), and the target height was 169.5 cm ( Figure 1 ).

**Figure 2:**
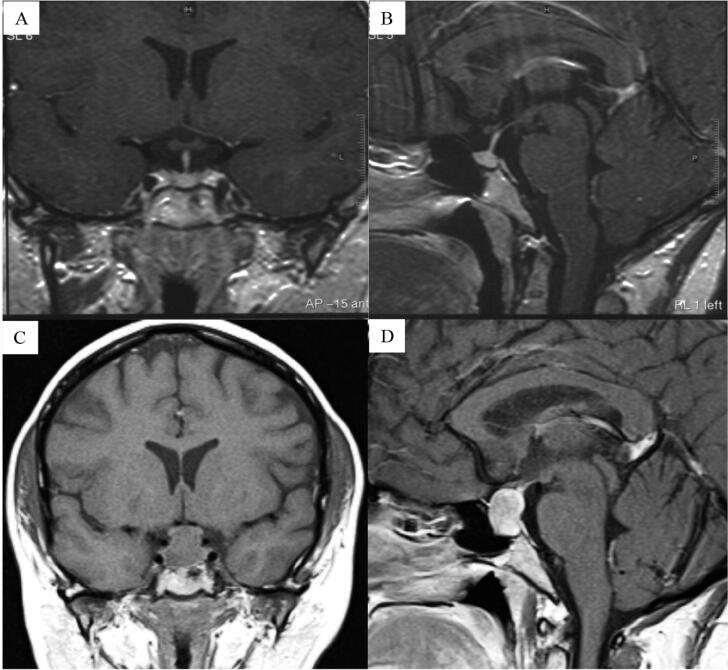
( **A, B** ) A magnetic resonance image (MRI) in T1 post-contrast of the patient’s pituitary shows an intact pituitary stalk and normal adenohypophysis. Five years later, the pituitary MRI in coronal section in T1 before contrast ( **C** ) and in sagittal section in T1 post-contrast ( **D** ) shows a macroadenoma measuring about 2.0 × 1.7 × 1.3 cm and compressing the optic chiasma. The posterior pituitary lobe and the pituitary stalk are not visible.

GHD was confirmed in 2007, when the patient was 28 years old, by a peak GH of 0.1 ng/mL during insulin-induced hypoglycemia (blood sugar = 36 mg/dL) ( Table 1 ). Insulin-like growth factor-1 (IGF-1) was < 25 ng/mL. At that time, magnetic resonance imaging (MRI) of the pituitary region showed normal adenohypophysis and intact pituitary stalk ( Figure 2 ). The molecular diagnosis of the index patient was previously described by Lido and cols. ( 13 ), with the allelic variant c.291+1G> T in the *GH1* gene in heterozygosis by Sanger sequencing. The patient’s mother was studied, and no mutation was found. The patient’s father was not available for study. The patient does not have brothers of the same father and mother. However, the other children of the patient’s father have normal stature.

**Table 1:** Combined hormone test, performed in 2007, when the patient first arrived at our service

Time (min)	-30	0	15	30	45	60	90
Glucose (mg/dL)	73	68	46	57	65	36	69
**GH** * (NV = up to 4.4 ng/mL)	0.1	0.1	0.1	0.1	0.1	0.1	0.1
**F** ** (NV = 5.4-25 ug/dL)	26.9	24.8	23.5	26	28.5	25.5	31.6
**TSH** (NV = 0.4-4.5 µU/mL)	4.8	4.6	36	34	26	18	16
**LH** (NV1.1-6.3 IU/L)	<0.6	<0.6	1.3	1.3	1.2	0.9	0.9
**FSH** (NV = 1.4-5.7 IU/L)	1.4	1.2	2.4	2.6	2.6	2.7	2.8
**PRL** (NV = 2-15 ng/mL)	19.3	12.2	77	64	53	28	108

Note: The patient was using oral contraceptives at the time these measurements were taken.

* NV = if hypoglycemia, GH > or = to 3.3 if glucose < 40 mg/dL.

** NV = if hypoglycemia, F > or = to 18 if glucose < 40 mg/dL.

GH: growth hormone; F: cortisol; TSH: thyroid-stimulating hormone; LH: luteinizing hormone; FSH: follicle-stimulating hormone; PRL: prolactin; NV: normal value

After rhGH therapy was introduced when the patient was 28 years old, central hypothyroidism was diagnosed by decreased free thyroxine (T4; 0.68 ng/dL, reference value [RV] = 0.7-1.4 ng/dL), slightly increased thyroid-stimulating hormone (TSH; 4.8 μU/mL, RV = 0.40-4.50 μU/mL), and negative anti-thyroid antibodies. Levothyroxine 50 µg per day was added to GH replacement. The patient was on rhGH and levothyroxine therapy for 5 years. In 2012, when the patients was 33 years old, a cortisol profile was requested due to a progressive increase in body weight (approximately 18 kg in 5 years), and hypercortisolism was confirmed with increased urinary and salivary cortisol levels ( Table 1 ). Besides increased BMI and blood pressure measures, no other clinical features of hypercortisolism, such as facial plethora, moon face, bruises, or filled supraclavicular fossa were present. An MRI of the sella turcica detected an expansive sellar-suprasellar lesion with lobulated contours, measuring about 2.0 × 1.7 × 1.3 cm. The lesion had shifted the optical chiasm and compresses the floor of the third ventricle ( Figure 2 ). At the time of the diagnosis of the pituitary macroadenoma, rhGH therapy was suspended, and the patient underwent total resection of the pituitary adenoma. Anatomopathological examination and immunohistochemistry of the surgical specimen confirmed an ACTH-secreting pituitary macroadenoma. ACTH was diffusely distributed in the tumor (over 50% of the cells expressed ACTH) ( Figure 1 ) and other hormones, such as prolactin, GH, TSH, follicle-stimulating hormone (FSH), and luteinizing hormone (LH) were not immunoexpressed. Eight years after surgery, the patient remains in remission, with no new evidence of tumor or hypercortisolism. All tests that analyze the cortisol profile remain negative for hypercortisolism in our investigation nowadays. However, she persists with GHD and central hypothyroidism. All clinical and laboratory data are described in Table 2 .

**Table 2:** Clinical and laboratory data before recombinant human growth hormone (rhGH) replacement (2007), 5 years after rhGH replacement with evidence of corticotropinoma (2012), and 1 and 5 years after tumor removal surgery (2014, 2018)

Years	2007	2012	2014	2018
Age (years)	28	33	35	37
Observation	No rhGH, no evidence of corticotropinoma	Under rhGH, with evidence of corticotropinoma	One year after corticotropinoma removal	Five years after corticotropinoma removal
BMI (kg/m²)	30.4	40.2	35.5	36.3
Weight (kg)	55.6	73.5	64.9	66.4
rhGH replacement	NO	YES	NO	NO
T4 replacement	NO	YES (100 µg)	YES (112.5 µg)	YES (112.5 µg)
Oral contraceptive use	YES	YES	YES	YES
Systolic blood pressure (mmHg)	120	137	103	NA
Diastolic blood pressure (mmHg)	80	95	63	NA
Fasting blood sugar (NV = 70-100 mg/dL)	70	79	69	73
IGF-1 (NV = 117-321 ng/mL)	<25	77	NA	35
Total cholesterol (NV = <190 mg/dL)	165	212	NA	164
LDL-cholesterol (NV = <100 mg/dL)	54	84	NA	71
HDL-cholesterol (NV = >60 mg/dL)	95	111	NA	80
Triglycerides (NV = <200 mg/dL)	78	83	NA	52
TSH (NV = 0.40-4.50 μU/mL)	4.6	0.37	NA	NA
Free T4 (NV = 0.7-1.4 ng/dL)	1.0	1.53	1.1	1.6
Prolactin (NV = 2-15 ng/mL)	12.2	30.6	18.2	10.9
ACTH (NV = <46 pg/mL)	NA	103	21	38.4
Plasma F (NV = 5-25 ug/dL)	24.8	21.3	20.7	11.5
Midnight salivary F (NV = <0.12 ug/dL)	NA	0.29	0.05	<0.170
24-h urinary free F (NV = 50-310 ug/24h)	NA	376	59.27	NA
F level after 1 mg dexamethasone suppression test (NV = <1.8 ug/dL)	NA	NA	1.3	<1.3

BMI: body mass index; GH: growth hormone; T4: thyroxine; TSH: thyroid-stimulating hormone; ACTH: adrenocorticotropic hormone; F: cortisol; NV: normal value; NA: not available

Knowing that mutation in *USP8* is one of the most common causes of ACTH-producing pituitary micro/macroadenomas, we analyzed the tumor using Sanger sequencing; we found a pathogenic variant in heterozygosis in *USP8* (c.2159C>G/p.Pro720Arg). This variant was not present in the DNA collected from the patient’s peripheral whole blood, confirming that this was a somatic variant.

## DISCUSSION

To the best of our knowledge, we describe the first case in the literature of a patient with GHD and central hypothyroidism who simultaneously presented hyperfunction of corticotrophs leading to Cushing disease. Lido and cols. ( 13 ) reported this patient as having IGHD, but during follow up under rhGH therapy, hypothyroidism was noted. It is important to note that under GHD, the conversion from T4 to T3 is decreased and TSH deficiency can be noted after rhGH therapy is started ( 14 ). However, there are reports of several patients with variants that result in skipping of exon 3 of *GH1* progress to CPHD ( 15 ), which was observed in the present case.

Specific mutations in the *GH1* gene at the splice site, in the exon splice enhancer, and intronic deletions, are responsible for the dominant form of IGHD (type II, OMIM 173100) ( 16 ). Point mutations located in the first six base pairs of the 5’ region of the *GH1* intron 3 result in the loss of exon 3 in the mutated allele. This leads to the expression of the 17.5 kDa GH isoform, which has a dominant-negative effect on the GH bioactive isoforms ( 17 ). The 17.5 kDa isoform is initially retained in the endoplasmic reticulum, disrupts the Golgi apparatus, and impairs the secretion of GH and other pituitary hormones, such as TSH, ACTH, and gonadotropins in humans ( 15 , 18 ), as well as in transgenic mice ( 19 ).

The allelic variant presented by the index patient in *GH1* (c.291+1G>T) was first described in the literature in 2006, in two different patients with IGHD type II ( 20 ). In 2010, Alatzoglou and cols. ( 10 ) reviewed 13 different splice site mutations in *GH1* ; two of these mutations are in the same position as the one presented by our index patient (IVS3+1G>A and IVS3+1G>C). Patients with type II IGHD show wide variability regarding the age of onset and severity of IGHD ( 21 ). They present with low but detectable GH levels combined with pathologically low IGF-1 concentrations, a variable height deficit (depending on the *GH1* gene alteration, there is a clinical variability in the severity of the IGHD II phenotype), and may or may not have anterior pituitary hypoplasia on MRI (38%-50%). The median age of diagnosis in patients with splice site mutations is 3 years old ( 22 ). To verify whether a similar event occurred in other patients of our cohort with CPHD, we sequenced *GH1* in 44 patients with patent pituitary stalk and a topic or non-visualized posterior lobe on MRI. We did not find any other pathogenic *GH1* allelic variants.

The *USP8* gene regulates epidermal growth factor receptor (EGFR) signaling by controlling its deubiquitination. Gain-of-function *USP8* somatic mutations in corticotropinomas increase its deubiquitinating effect and thus overall EGFR signaling activation, leading to enhanced proopiomelanocortin (POMC) expression and ACTH secretion. Notably, all *USP8* somatic mutations associated with Cushing disease are located in the 14-3-3 binding motif (all mutations are located between amino acids 713 and 720, close to the protein’s catalytic domain) ( 23 , 24 ). These findings are the result of whole exome analyses of corticotropinomas removed from the pituitary of patients with Cushing disease. The most frequent mutations were p.Ser718Pro, p.Ser718del, and p.Pro720Arg. These mutants completely lose the capability to bind to 14-3-3 proteins, showing higher deubiquitinase catalytic domain (DUB) activity than the wild type counterpart. Further analyses revealed that all these *USP8* mutants underwent proteolytic cleavage, giving rise to two fragments (about 90 and 40 kDa, respectively). The latter fragment contains the complete DUB sequence, but it lacks control of the 14-3-3 proteins, thus acquiring elevated DUB activity. Transfection of USP8 mutants in cultured cells decreases the levels of EGF-induced EGFR ubiquitination. Moreover, USP8 mutants induce both EGFR retention on the plasma membrane and recycling of endocytosed EGFR back to the cell surface, causing continuous activation of EGF signaling. EGFR is deeply involved in ACTH-producing pituitary neuroendocrine tumors (PitNETs): It represents a strong promoter of ACTH synthesis ( 25 ).

In a screen of 108 ACTH-secreting pituitary adenomas, Ma and cols. ( 26 ) identified 17 different heterozygous somatic mutations in the *USP8* gene, including the c.2159C>G somatic mutation, resulting in p.Pro720Arg. This mutation is one of three most recurrent mutations that, together, account for the vast majority of the *USP8-* mutated cases (over 77% of the *USP8* -mutated tumors). *USP8* mutations have been identified in both female and male patients, but their frequency is significantly higher in female (67.7%) compared with male (38.1%) patients ( 26 ). Most *USP8* -mutated pituitary adenomas are small in size (<0.5 cm) and diffusely distributed within the sella turcica. Patients with mutated *USP8* show comparable levels of plasma ACTH, midnight serum cortisol, and 24-h urinary free cortisol to patients with wild type *USP8* . ACTH-secreting pituitary adenomas (PAs) with mutated USP8 display a higher incidence of EGFR expression as well as elevated EGFR protein abundance and expression of *POMC* mRNA, which encodes the precursor of ACTH. PAs with mutated *USP8* are significantly smaller in size and have higher ACTH production than wild type PAs ( 26 ). However, the authors of a Brazilian study observed a tendency toward more somatic *USP8* mutations in macrocorticotropinomas than in microcorticotropinomas ( 27 ). Until now, only one case of USP8 germline mutation has been described in the literature ( 24 ).

It is important to note that the index patient presented a very mild Cushing disease phenotype with only weight gain and the absence of any other signs of hypercortisolism, such as facial plethora, moon face, bruises, or filled supraclavicular fossa. It is known that tumor size does not directly correlate with the extent of hormonal activity in ACTH-secreting adenomas ( 28 ). The patient has maintained persistent remission of hypercortisolism 8 years after surgery. A high recurrence rate has been reported in patients harboring a *USP8* mutation in the follow-up and up to 10 years later ( 29 ). Therefore, continuous vigilance is necessary in the patient in the present study. Although we could hypothesize an effect of rhGH replacement on the tumor growth, based on our literature search, we could not demonstrate a relationship between the GH and USP8 (or EGFR) signaling pathways.

In conclusion, the present study has shown that a point mutation leading to type II GHD is a rare event in our cohort but when present may progress to CPHD. There was a rare coincidence of a germline variant in *GH1* and a somatic variant in *USP8* , which were responsible for hypopituitarism and Cushing disease in the same patient.
